# An Unusual Presentation of Multiple Myeloma: A 71-Year-Old Female With a Single Lytic Lesion of Her Appendicular Skeleton

**DOI:** 10.7759/cureus.24725

**Published:** 2022-05-04

**Authors:** Ahamed Khalyfa, Alessandra C Carrillo, Yhana Chavis

**Affiliations:** 1 Internal Medicine, Franciscan Health, Olympia Fields, USA

**Keywords:** rank and rankl, pathologic fracture, crab, solitary lytic lesion, diagnosis of multiple myeloma

## Abstract

Multiple myeloma is a devastating illness with a hallmark of end-organ damage. The clinical presentation of multiple myeloma often includes the involvement of CRAB (hypercalcemia, renal failure, anemia, bone lesions) symptoms. We present a case of a patient who did not exhibit the typical presentation of multiple myeloma making her case unique and her diagnosis more difficult. In addition to the CRAB criteria, typical symptomatology includes constipation, pain, fatigue, and peripheral sensory issues. The purpose of this case report is to bring awareness to both multiple myeloma and this particular presentation.

The patient is a 71-year-old female with a past medical history of hypertension, hypothyroidism, and rheumatoid arthritis who presented with a chief complaint of right shoulder pain. The patient’s initial labs were significant for a total protein of 9.3, albumin of 3.4, corrected calcium of 9.3, hemoglobin 10.6 (with baseline near 11-12), and creatinine of 1.0 (baseline of 1.0). The patient’s right upper extremity X-rays were significant for a right humeral fracture. The patient had a serum kappa/lambda ratio of 15.94. Bone marrow biopsy revealed 50% kappa-restricted cells, consistent with a diagnosis of multiple myeloma. The patient’s subsequent bone survey and CT scan were negative for any additional lesions. The patient had subsequent radiation therapy followed by maintenance therapy with bortezomib, lenalidomide, and dexamethasone with improvement in her symptoms.

MM is a complex pathophysiological disease and equally as complex in diagnosis as the presentation is varied and sometimes obscure as noted in the case presented here. Although bone lytic lesions are part of the CRAB criteria, it is rare for them to present in patients with MM in an isolated manner with no corresponding lab abnormalities. With this case, we aim to shed light upon an atypical presentation of MM, notably one that solely involves a pathological fracture in a non-axial distribution.

## Introduction

Multiple myeloma (MM) is a devastating hematologic malignancy that accounts for approximately 10% of all heme malignancies, making it the second most common hematologic malignancy [1-2]. It belongs to a family of diseases known as plasma cell dyscrasias [2]. The plasma cell dyscrasias include monoclonal gammopathy of undetermined significance (MGUS), smoldering multiple myeloma (SMM), and malignant multiple myeloma [2]. The relationship between the plasma cell dyscrasias is deeply intertwined with almost all cases of MM developing from MGUS [3-4]. They develop into other lymphoproliferative disorders, amyloidosis, or Waldenstrom’s macroglobulinemia [2]. The disease is devastating for those who develop malignant MM, causing significant end-organ damage. Early recognition, surveillance of MM precursors, and prompt initiation of treatment are vital to the patient’s overall survival.

## Case presentation

**Figure 1:**
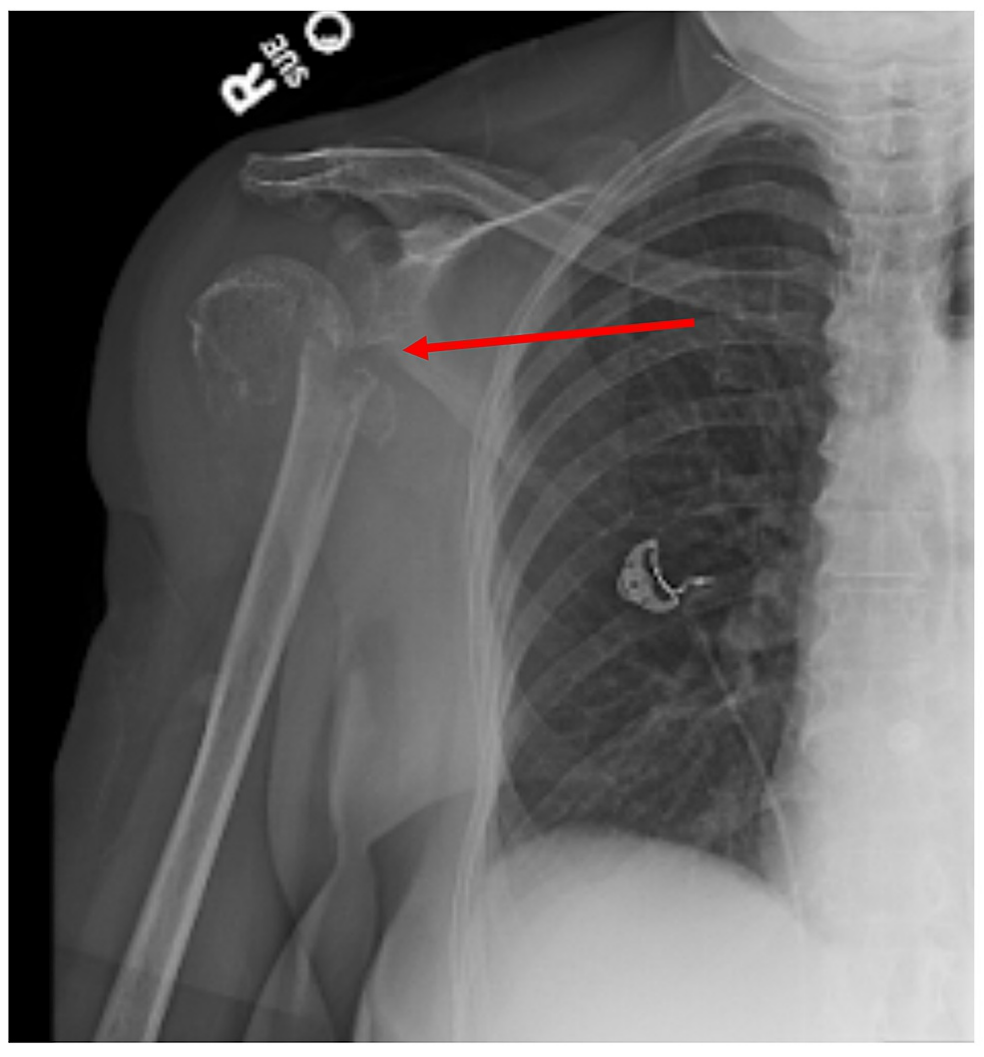
Right upper extremity X-ray depicting a right humeral neck fracture (red arrow)

The patient was a 71-year-old female with a past medical history of hypertension, hypothyroidism, asymptomatic rheumatoid arthritis (seropositive but in remission since 2007, on methotrexate for one year but off medications for 15 years), and vertigo with complaints of right shoulder pain. The patient was attempting to open an aluminum can when she heard a “pop” in her right shoulder. She had no prior history of fractures or a family history of fractures. Her medications were amlodipine, spironolactone, tramadol, levothyroxine, and meclizine. The patient had no prior history of osteoporosis and her previous DEXA (dual-energy X-ray absorptiometry) scan approximately three years prior to admission revealed a T score of -0.4. Laboratory values were significant for a total protein of 9.3, albumin of 3.4, corrected calcium of 9.3, hemoglobin 10.6 (with baseline near 11-12), and creatinine of 1.0 (baseline of 1.0) (Table 1). Urine immuno-electrophoresis showed no evidence of monoclonal paraproteins. The patient’s right upper extremity X-rays were significant for a right humeral fracture (Figure 1).

**Table 1:** Patient's initial laboratory values

Lab	Laboratory results	Reference values
Calcium	9.3 mg/dl	8.6-10.3 mg/dl
Albumin	3.6 g/dl	4.0 g/dl
Hemoglobin	10.6 g/dl	12-15 g/dl
Total protein	9.3 g/dl	6.4-8.9 g/dl
Creatinine	1 mg/dl	0.6-1.2 mg/dl
Serum kappa/lambda ratio	15.94 mg/L	0.26-1.64 mg/L
Beta 2 microglobulin	0.230 mg/dl	0.097-0.184 mg/dl
Immunoglobulin G	4172 mg/dl	600-1500 mg/dl
Immunoglobulin A	23 mg/dl	50-400mg/dl
Immunoglobulin M	234 mg/dl	45-281 mg/dl

Given the mechanism of fracture, the patient was worked up for pathologic fracture and was found to have a serum kappa/lambda ratio of 15.94. She was evaluated by orthopedic surgery and treated conservatively with a sling given her age. In addition, a bone marrow biopsy showed 50% kappa-restricted cells, consistent with a diagnosis of multiple myeloma. The patient had a subsequent bone survey and CT scan without evidence of other lesions noted. The patient had subsequent radiation therapy followed by maintenance therapy with bortezomib, lenalidomide, and dexamethasone with improvement in her symptoms.

## Discussion

This case was specifically chosen for review, as it is a novel presentation of multiple myeloma given this patient presented with a pathologic fracture, specifically of her right upper extremity. She only had one lesion throughout her entire skeletal structure. Initial presentation for these patients varies depending on the severity of their disease. Pathologic fractures occur in patients with multiple myeloma, but they are more common in the axial skeleton and more specifically the vertebral column and the ribs [5]. Melton et al. conducted an analysis of the fracture risk following a diagnosis of MM. They concluded that patients with MM were at an increased risk for fractures compared to the adult population without MM. Specifically, myeloma patients experience a nine-fold increase in fracture risk [5]. Of those fractures, 69% resulted from pathological lytic lesions [5]. Almost all the lesions occurred in the axial skeleton exclusively [5]. By their analysis, the relative risk of any axial fracture was 14 (95% CI 11-17) as compared to the appendicular skeleton 2.0 (95% CI 1.2-3.0) [5]. Additionally, CRAB (hypercalcemia, renal failure, anemia, bone lesions) symptoms are also well-described in multiple myeloma and include hypercalcemia, renal insufficiency/failure, anemia, and bone pain. According to the Revised International Myeloma Working Group diagnostic criteria for multiple myeloma and smoldering myeloma, myeloma defining events entail the following: Hypercalcemia: serum calcium >0.25 mmol/L (>1 mg/dL) higher than the upper limit of normal or >2.75 mmol/L (>11 mg/dL); Renal insufficiency: creatinine clearance <40 mL per min or serum creatinine >177 μmol/L (>2 mg/dL); Anemia: hemoglobin value of >2 g/dL below the lower limit of normal or a hemoglobin value <10 g/dL; Bone lesions: one or more osteolytic lesions on skeletal radiography, CT, or positron emission tomography (PET)-CT (Table 2) [6].

**Table 2:** International Myeloma Working Group Diagnostic Criteria for Multiple Myeloma Source: [6] CT: Computed Tomography, PET: Positron Emission Tomography, MRI: Magnetic Resonance Imaging, FLC: Free Light Chain, IgG: Immunoglobulin G, IgA: Immunoglobulin A

Criteria	
Multiple Myeloma	
Both criteria must be met:	
1. Clonal bone marrow plasma cells >/= 10% or biopsy-proven bony or extramedullary plasmacytoma	
2. Any one or more myeloma defining events:	
Evidence of end-organ damage that can be attributed to the underlying plasma cell proliferative disorder, specifically: a. Hypercalcemia: serum calcium > 0.25 mmol/L (> 1 mg/dL) higher than the upper limit of normal or > 2.75 mmol/L (> 11 mg/dL). b. Renal insufficiency: creatinine clearance < 40 mL/min or serum creatinine > 177 µmol/L (>2 mg/dL). c. Anemia: hemoglobin value of >2 g/dL below the lower limit of normal or a hemoglobin value < 10 g/dL. d. Bone lesions: one or more osteolytic lesions on skeletal radiography, CT or PET-CT	
Clonal bone marrow plasma cell percentage ≥ 60%	
Involved: uninvolved serum FLC ratio ≥ 100 (involved FLC level must be ≥100 mg/L)	
>1 focal lesion on MRI studies (at least 5 mm in size)	
Smoldering myeloma	
Both criteria must be met:	
1. Serum monoclonal protein (IgG or IgA) >/= 3 gm/dL or urinary monoclonal protein >/= 500 mg per 24h and/or clonal bone marrow plasma cells 10%- 60%.	
2. Absence of myeloma defining events or amyloidosis	

Our case is unique, as our patient did not have initial laboratory work consistent with the organ dysfunction that is a hallmark of MM. Furthermore, she only had one lytic lesion in a non-axial distribution. With respect to the prevalence of CRAB symptoms in myeloma patients, few studies have identified the answer to this question. One study that included 113 patients found that the frequency of CRAB symptoms among their study population was: hypercalcemia (6%), renal failure (29%), anemia (57%), and bone disease (68%) [7]. Another study comprising 170 patients showed that among patients with symptomatic MM, 74% presented with CRAB symptoms, 20% presented with non-CRAB manifestations, and 6% had evidence of both clinical features [8]. Among patients with non-CRAB manifestations, symptomatology was identified as neuropathy, extramedullary involvement, hyperviscosity syndrome, concomitant amyloidosis, hemorrhage/coagulopathy, fever or weight loss, primary plasma cell leukemia, infections, cryoglobulinemia, or gout [8]. Interestingly, our patient did not exhibit these non-CRAB symptoms as well, adding to the uniqueness of her clinical case. Although these studies are underpowered, they convey the notion that among patients with symptomatic MM, a majority of them should present with CRAB symptoms.

The pathophysiology of bony destruction involves the disruption of homeostatic bone remodeling between osteoclasts and osteoblasts [9]. Several molecular pathways, such as RANK/RANKL, Notch, and Wnt, have been identified as key players in favoring an osteolytic environment [10]. Other macrophage inflammatory proteins, (MIP)-1alpha and MIP-1beta, have also been identified to be key mediators in creating bone resorption [11]. It is well-known that multiple myeloma cells secrete RANKL, which binds RANK and leads to the subsequent development of bone resorption. Plasma cells also have been shown to secrete PTHrP, which leads to increased RANKL expression [11]. Furthermore, multiple myeloma cells also activate cascades that lead to decreased OPG expression, which is closely linked to bone lysis [12].

## Conclusions

MM is a complex pathophysiological disease and is equally as complex in diagnosis, as the presentation is varied and sometimes obscure as noted in the case presented here. Although bone lytic lesions are part of the CRAB criteria, it is rare for them to present in patients with MM in an isolated manner with no corresponding lab abnormalities. With this case, we aim to shed light upon an atypical presentation of MM, notably one that solely involves a pathological fracture in a non-axial distribution.
